# Discriminant validity, responsiveness and reliability of the rheumatoid arthritis-specific Work Productivity Survey (WPS-RA)

**DOI:** 10.1186/ar2702

**Published:** 2009-05-20

**Authors:** Jane T Osterhaus, Oana Purcaru, Lance Richard

**Affiliations:** 1 Wasatch Health Outcomes, 2613 Silver Cloud Drive, Park City, UT 84060, USA; 2 Global Health Outcomes Research, UCB Pharma, Chemin du Foriest, 1420 Braine-l'Alleud, Belgium; 3 Global Health Outcomes Research, UCB Pharma, 208 Bath Road Slough, Berkshire SL1 3WE, UK

## Abstract

**Introduction:**

The rheumatoid arthritis-specific Work Productivity Survey (WPS-RA) measures the impact of rheumatoid arthritis (RA) and treatment on patient productivity within and outside the home. It contains nine questions addressing employment status, productivity within and outside the home, and daily activities. The objective of this paper was to evaluate the discriminant validity, responsiveness, and reliability of the WPS-RA in patients with active RA.

**Methods:**

Two hundred twenty subjects (mean age was 53.8 years, 83.6% were female, mean disease duration was 9.54 years, mean number of disease-modifying anti-rheumatic drugs failed was 2, and 38.6% were employed outside the home) in a phase III, 24-week, double-blind, placebo-controlled trial completed the WPS-RA at baseline and every 4 weeks until withdrawal/study completion. Validity was evaluated via known groups using baseline data (first and third quartiles of subjects' Health Assessment Questionnaire – Disability Index [HAQ-DI] scores and Short Form-36 health survey [SF-36] scores). To evaluate responsiveness, mean changes in WPS-RA at week 24 were compared between American College of Rheumatology 20% improvement criteria (ACR20) (or HAQ-DI) responders and non-responders. Standardized response mean (SRM) was also used to quantify responsiveness. All group comparisons were conducted using a non-parametric bootstrap-t method.

**Results:**

Subjects with lower HAQ-DI or SF-36 scores generally had statistically greater RA-associated losses in productivity within and outside the home compared with subjects with higher scores (25 of 32 evaluations were statistically significant). Smallest differences between groupswere seen in work absenteeism and days with outside help. At week 24, ACR20 and HAQ-DI responders reported large improvements in productivity within and outside the home; non-responders reported mainly a worsening in productivity (*P *≤ 0.05). Effect size for productivity changes in ACR20 or HAQ-DI responders was moderate to large for six out of eight items (SRM = 0.48 to 1.12). The effect size was small for work absenteeism and days with outside help. (SRM = 0.4 and 0.24, respectively). In non-responders, the magnitude of change was negligible (SRM < 0.1) or small (SRM < 0.3).

**Conclusions:**

The WPS-RA has demonstrated properties of discriminative validity, reliability, and responsiveness for the measurement of productivity within and outside the home in subjects with active RA.

## Introduction

Rheumatoid arthritis (RA) places an exceptionally high burden on society. This is because the disease's impact on functioning and the average age at onset occur during what would typically be an individual's peak working years. While the direct costs of RA are notable (estimated to be as high as US $5.5 billion), the indirect costs of RA associated with paid and unpaid (household) work are generally estimated to be significantly higher due to high levels of disability (estimated to be as high as US $10.2 billion) [1].

There has been a lot of research published about the impact of RA on a person's ability to carry out paid work [2-5]. Patients with arthritis have higher unemployment rates than those with other chronic diseases and have more time lost from work [6-8]. Ability to work tends to decline as duration of RA, physical disability, and age increase [3,9,10]. Physical functioning, as measured by the Health Assessment Questionnaire – Disability Index (HAQ-DI), has been associated with the ability to work [11-13] but does not fully address the impact of RA on one's ability to perform work-related tasks, whether related to paid work or household tasks.

Research on unpaid work outcomes has not been as prevalent, although the importance of understanding this area has been noted. Mittendorf and colleagues [14] reported that, during a clinical trial treatment period of up to 3 years, the percentage of patients with long-standing and severe RA receiving personal help ranged from 40.8% at baseline to 48.7% at study end. Patients received the greatest degree of personal help for household tasks, followed by help for personal care. With the exception of child care, the majority of personal help was provided free of charge [14].

Verstappen and colleagues [4] reported on the household productivity costs of a sample in The Netherlands using the Utrecht RA Cohort. The cohort consisted of patients with RA at all stages of the disease. Household productivity losses were defined as housekeeping tasks that had to be carried out by formal (paid) or informal (unpaid) caregivers if the patient was unable to perform the tasks because of RA. Some form of household help was needed by 51% of patients, including 12% who required formal assistance and 15% who required private help. Females tended to require more help than males, as did those individuals with greater disability [4]. While these reports provide documentation of the burden of RA on paid and unpaid work, a challenge lies in how best to measure these outcomes and to report the impact of RA interventions in reducing the work limitations due to the disease.

Traditionally, productivity has been assessed in the workplace. However, there is an increasing awareness of the potential impact of RA on productivity within the home. Workplace productivity is often described in terms of efficiency and output of the workplace. Worker productivity, or work productivity as it is most commonly called, is a critical part of that broader measure of workplace productivity. It is the component that is directly affected by an illness and potentially amenable to health-related interventions [15]. Worker productivity is generally subdivided into two distinct states: absenteeism and presenteeism. Absenteeism, or absence from work, is generally defined as work days missed due to health problems, and presenteeism refers to reduced performance or productivity due to health reasons while at work [15].

A plethora of measures have been used in various settings to specifically measure the impact of RA on work absence and work productivity. Escorpizo and colleagues [15] recently reviewed the measures of work productivity and their relevance to RA. The authors note that there is not yet a gold standard measure for assessing productivity in RA, but the importance of measuring it is agreed upon. The challenge is how to appropriately measure the time, resources, or units of lost effort associated with RA.

Most existing measures attempt to capture lost paid work days and some measure of the impact of working with symptoms. However, current measures tend to ignore productivity issues within the home and participation in social activities. A notable proportion of people with RA drop out of the workforce due to their disability, and yet they still need to do work around the house or someone has to do it for them; therefore, the impact of the condition on unpaid work also warrants consideration [10].

Consequently, a survey that would measure both absenteeism and work productivity in RA patients was developed for use in clinical trials. The survey, the RA-specific Work Productivity Survey (WPS-RA), was designed to estimate the productivity limitations associated with RA on paid jobs outside the home, on unpaid work within the home, and on other social activities during the preceding month. The questionnaire was developed by reviewing the RA literature as well as that of other chronic conditions in which work productivity has been previously explored, documented, and captured (for example, migraine headache and depression). Since the questionnaire was intended to be relevant for all patients, specific aspects of work were not addressed (for example, we did not ask about the ability to lift heavy objects). The goal was to obtain an estimate of the amount of time the respondent missed work or other activities or was less functional at work or other activities due to their RA. The survey items were framed based around work outside the home (paid work) as well as inside the home (unpaid work) and other activities that might be limited due to RA and/or its treatment. Items were selected based on the desire not to overburden patients with too many questions but to efficiently capture information that might be of use to health care professionals and payers in making treatment decisions regarding RA interventions. The actual concepts captured by the items created are fairly straightforward. It was presumed that the patients would not have major problems with these concepts, which generally focus on quantitative issues (for example, days of work missed and days of social activities missed).

The objective of this paper was to evaluate the disciminant validity (that is, the ability to differentiate between patients with different RA symptom severity), responsiveness to clinically meaningful changes, and reliability of the WPS-RA. The survey was intended to capture the patient's perspective of aspects of work (within and outside the home) that are difficult and that change over time due to disease progression or to clinical interventions.

## Materials and methods

### Subjects and study design

Subjects for this study were enrolled in the FAST 4WARD (efficacy and safety of certolizumab pegol monotherapy every 4 weeks dosage in rheumatoid arthritis) study, a 24-week, multicenter, randomized, double-blind, placebo-controlled clinical trial of certolizumab pegol 400 mg or placebo, conducted at 36 sites in three countries (Austria, the Czech Republic, and the US) from June 2003 to July 2004. Institutional review boards or ethics committees approved the protocol at each center. All patients gave written consent, and the study was conducted in accordance with the Declaration of Helsinki. Certolizumab pegol is the only PEGylated anti-tumor necrosis factor for the treatment of RA. It has been studied in three phase III trials, showing efficacy as a combination therapy to methotrexate and monotherapy [16-18].

Patients were randomly assigned 1:1 to receive lyophilized subcutaneous certolizumab pegol 400 mg or placebo (sorbitol) every 4 weeks (q4w) from baseline to week 20. Patients who completed the study or withdrew on or after week 12 were eligible and encouraged to enter an open-label study of certolizumab pegol 400 mg q4w (unless withdrawn due to non-compliance or possible treatment-related adverse events). Patients who withdrew after taking at least one study dose were asked to return for an early-withdrawal visit.

The primary efficacy endpoint was the American College of Rheumatology 20% improvement criteria (ACR20) response at week 24 [19,20]. Secondary endpoints included physical functioning, assessed using the HAQ-DI, and health-related quality of life (HRQoL), assessed using the Short Form 36 health survey (SF-36) and the WPS-RA. Efficacy assessments (ACR and HAQ-DI) were conducted at weeks 0, 1, 2, and 4 and then q4w until the end of the study or withdrawal; the SF-36 was administered at weeks 0, 4, and 12 and at the end of the study or withdrawal; and the WPS-RA was administered at weeks 0 and 4 and then q4w until the end of the study or withdrawal.

### Questionnaires

The WPS-RA is a disease-specific questionnaire assessing the impact of RA on productivity within and outside the home and daily activities during the preceding month. It is self-reported by the patient, is interviewer-administered, and has a 1-month recall period (Additional data file 1).

One item of the WPS-RA addresses current labor market participation (that is, 'are you currently employed outside the home?'). This is a strong indicator of work ability because not working implies complete loss of paid productivity. There are also normative and comparative data available on employment status. Two items capture self-reported work absences due to arthritis, and two items capture the same concept but applied to non-paid work. These are separated into full and partial days (that is, days of work missed and days with productivity reduced by at least half). Additional items capture the respondent's estimate of the extent to which arthritis has interfered with the patient's work productivity (paid and non-paid) on a scale of 0 to 10 (0 = 'no interference' and 10 = 'complete interference'), the number of days in the last month outside help was hired because of arthritis, and the number of days in the last month family, social, or leisure activities were missed because of arthritis.

The HAQ-DI is a patient-reported questionnaire that provides an assessment of the impact of the disease on physical function and disability [21]. The HAQ-DI contains 20 items divided into 8 domains that measure dressing and grooming, arising, eating, walking, hygiene, reach, grip, and common daily activities. Patients are required to indicate the degree of difficulty they have experienced in each domain in the past week on a 4-point scale that ranges from 0 (without difficulty) to 3 (unable to do). The highest score in each category is then summed (0 to 24) and divided by the number of categories scored to give a disability index that ranges from 0 to 3. HAQ-DI scores of 0 to 1 generally represent mild to moderate functional difficulty, 1 to 2 represent moderate to severe functional difficulty, and 2 to 3 indicate severe to very severe functional limitations or disability [22].

In this study, a meaningful improvement from baseline in physical functioning was assessed using a minimum clinically important difference (MCID) for a change in the HAQ-DI score. An MCID in the HAQ-DI score has been reported to be 0.22 on the 0-to-3 scale in general samples of RA patients [23,24].

The SF-36 is a widely used generic HRQoL instrument that evaluates eight health domains: physical functioning, role physical, bodily pain, general health, vitality, social functioning, role emotional, and mental health [25]. The eight domains are summarized in two component summaries: the Physical Component Summary (PCS) and the Mental Component Summary (MCS) [26]. Scores for the SF-36 range between 0 and 100, with higher scores indicating a better HRQoL.

The ACR 20/50/70 response assesses the treatment of symptoms and signs in subjects with active RA. Based on the ACR Core Set of Response Criteria for Rheumatoid Arthritis Clinical Trials, a subject is defined as an ACR 20/50/70 responder if there is an improvement (that is, reduction) of at least 20%/50%/70%, respectively, from baseline in the number of tender and swollen joints and in at least three of the five core set measures (Patient's and Physician's Global Assessments of Disease Activity – Visual Analog Scale [VAS], Patient's Assessment of Arthritis Pain – VAS, an acute-phase reactant [C-reactive protein was used], and physical functioning based on the HAQ-DI) [19].

### Data handling and statistical analysis

The assessment of the psychometric properties (discriminant validity, responsiveness, and reliability) of the WPS-RA was performed on the overall modified intent-to-treat (mITT) population (that is, randomly assigned patients, who had taken at least one dose of study drug), regardless of the randomization group.

### Discriminant validity

The discriminant validity of the WPS-RA was assessed using the known-groups validation method. Patients with lower physical functioning or with lower HRQoL were expected *a priori *to have a reduced productivity within and outside the home compared with patients with a higher physical functioning or HRQoL, respectively.

For this purpose, the HAQ-DI and the SF-36 scores were considered as categorical variables and the known groups were formed using as cutoff points the baseline first and third quartile scores in HAQ-DI and SF-36 in the overall population. More specifically, we compared those patients with scores in the lowest 25th percentile to those with scores in the highest 25th percentile of the population. Based on her/his physical functioning score at baseline, a subject was considered as having either a 'best' (HAQ-DI score ≤ first quartile) or 'worst' (HAQ-DI score ≥ third quartile) physical functioning. Subjects with a 'best' HRQoL were those with a baseline SF-36 score ≥ third quartile, whereas those with a score ≤ first quartile were considered as having a 'worst' HRQoL.

The discriminant validity of the WPS-RA was assessed at baseline on observed data on all randomly assigned subjects (that is, the overall mITT population). To test the validity of productivity at paid work, the HAQ-DI and SF-36 cutoff points were computed only on the subjects employed outside the home, whereas for productivity within the home, the HAQ-DI and SF-36 thresholds were computed on all subjects. Secondary analyses were conducted using the eight SF-36 domain scores to confirm these analyses.

A non-parametric bootstrap-t method was used to compare the mean responses to the WPS-RA questions between the groups [27]. This method was favored because of the highly skewed distribution of the WPS-RA scores. Bootstrap analyses were performed with 4,000 replications. A variance-stabilizing transformation was used in order to adjust for dependence graphically observed between bootstrap values and the corresponding standard error.

### Responsiveness to clinical changes and reliability

The responsiveness to clinical changes of the WPS-RA was assessed at week 24 on the overall mITT population and was tested against two meaningful clinical changes in patients: the ACR20 and the physical functioning (HAQ-DI) response.

According to the primary analysis of the FAST 4WARD study, a patient was considered an ACR20 'responder' if he/she met the criteria of ACR20 improvement from baseline at week 24. Any patient who withdrew from the study at any time during the study for any reason or who did not meet criteria for ACR20 response at week 24 was considered a non-responder. Patients reporting a decrease from weeks 0 to 24 in the HAQ-DI score of at least 0.22, in absolute value, were considered HAQ-DI 'responders'. Any patient who did not fulfill this criteria or who withdrew from the study at any time during the study for any reason was considered a HAQ-DI non-responder.

Changes in WPS-RA responses from weeks 0 to 24 were compared between responders and non-responders (to ACR20 or HAQ-DI) using a non-parametric bootstrap-t method [27]. When the WPS-RA response of a subject was missing at week 24, the last available observation was carried forward provided that the ACR20 (or HAQ-DI) response status was known for week 24. Patients with an unknown response status were not considered in the analyses.

In addition, the standardized response mean (SRM) was computed for each WPS-RA question. The SRM is computed by dividing the mean change in score between two visits by the standard deviation of that change. The SRM is the most widely used measure of size, indicating whether a change was large relative to the variability of the measurements. Standard thresholds for the SRM (absolute values) have been proposed in order to interpret the size of the effects: 'small' between 0.2 and 0.5, 'moderate' from 0.5 to 0.8, and 'large' greater than 0.8 [28].

Reliability of the WPS-RA was tested in conjunction with the responsiveness by comparing the changes in WPS-RA responses in patients achieving an ACR20 response (or an HAQ-DI response) with the change in responses in patients not achieving an ACR20 response (or not achieving an HAQ-DI response) at week 24. The statistical analyses were performed using the SAS version 8.2 (SAS Institute Inc., Cary, NC, USA).

## Results

### Patients

At baseline, 220 patients with active RA were randomly assigned to certolizumab pegol 400 mg (n = 111) or placebo (n = 109), with 76 (68.5%) and 28 (25.7%) patients in each group, respectively, completing treatment at week 24.

### Completion rates of the WPS-RA at baseline

At baseline, all subjects completed the survey. Out of nine questions, six were answered by all of the subjects. For the other three questions, the percentage of missing responses was small, ranging from 0.45% to 1.82%, suggesting that patients had no difficulties with the items or their responses.

### Demographic and clinical characteristics

Baseline demographics and clinical characteristics of the randomly assigned patients are summarized in Table 1. The mean age (range) of the population at baseline was 53.8 (21 to 80) years. Subjects employed outside the home (38.6%), those work-disabled due to RA (20%), and homemakers (10.5%) had similar mean ages (49.03, 51.7, and 50.5 years, respectively). The average age for retired subjects (25%) was 66.2 years. Of the randomly assigned subjects, 83.6% were women. The mean disease duration was 9.52 years, with subjects having moderate to severe RA at enrollment.

**Table 1:** Demographic and clinical characteristics of randomly assigned patients (modified intent-to-treat population) at baseline

	All randomly assigned (n = 220)
Mean age (range), years	53.8 (21 to 80)
Female gender, number (percentage)	184 (83.6%)
Caucasians, number (percentage)	177 (80.5%)
Country, number (percentage)	
Austria	3 (1.4%)
Czech Republic	52 (23.6%)
United States	165 (75%)
Employment status^a^, number (percentage)	
Employed outside the home	85 (38.6%)
Homemakers	23 (10.5%)
Retired	55 (25.0%)
Unable to work due to arthritis	44 (20.0%)
Other	13 (5.9%)
Job function if employed^a^, number (percentage)	
Non-manual	41 (48.2%)
Manual with no supervisory duties	14 (16.5%)
Mixed (manual and non-manual)	30 (35.3%)
Mean duration of RA (SD), years	9.52 (8.93)
Mean age at RA onset (SD), years	44.31 (13.57)
Mean disease activity, DAS28(3) (SD)	6.31 (1.0)
DAS28(3) group with DAS28 of >5.1, number (percentage)	196 (89.1%)
Mean number of prior DMARDs (range)	2 (0 to 8)

^a^Captured by the Work Productivity Survey – Rheumatoid Arthritis (WPS-RA). DAS, disease activity score; DAS28, disease activity score using 28 joint counts; DMARD, disease-modifying anti-rheumatic drug; RA, rheumatoid arthritis; SD, standard deviation.

### Baseline productivity within and outside the home, physical functioning, and health-related quality of life

Baseline productivity, physical functioning, and HRQoL are summarized in Table 2. Among the employed subjects, 32.9% reported absenteeism (the interquartile range was 0 to 1.5 days missed), 58.8% presenteeism (interquartile range of 0 to 7 days), and 92.9% interference of RA with their productivity at work over the preceding month. Almost all patients reported missed days of household work (75%), days with productivity of less than or equal to 50% in household work (86.3%), and interference of the disease with their productivity at home (93.5%) over the past month. The rate of RA interference with household work was slightly higher than the rate of reported work interference; the mean for household productivity (5.8) was above the average rate of interference and the mean for work productivity (4.5) was below the average. Additionally, 56.8% had missed days of social activities and 18% had to hire outside help. On average, at baseline, subjects had moderate to severe physical disability (mean HAQ-DI of 1.5) and low physical HRQoL.

**Table 2:** Productivity, physical functioning, and health-related quality of life at baseline as assessed by WPS-RA, HAQ-DI, and SF-36

	All randomly assigned^a^ (n = 220)		
	
	Number	Mean (SD)	Median
	
WPS-RA^b^			
Number of days of work missed (absenteeism)	85	2.2 (5.85)	0
Number of days with productivity = 50% at work (presenteeism)	85	5.9 (8.56)	2
Rate of arthritis interference with work productvity^c^	85	4.5 (2.50)	5
Number of days of household work missed	220	9.2 (9.84)	5
Number of days with productivity = 50% in household work	219	11.2 (10.00)	10
Number of days of family, social, or leisure activities missed	220	4.0 (6.74)	2
Number of days with outside help	217	1.2 (4.29)	0
Rate of arthritis interference with household work productvity^c^	216	5.8 (2.75)	5.5
HAQ-DI	219	1.5 (0.64)	1.5
SF-36 PCS	216	27.88 (7.84)	27.51
SF-36 MCS	216	44.71 (11.46)	45.28

^a^Modified intent-to-treat population; ^b^Work Productivity Survey – Rheumatoid Arthritis (WPS-RA) recall period is 1 month; ^c^score on a scale of 0 to 10 points (0 = no interference and 10 = complete interference). HAQ-DI, Health Assessment Questionnaire – Disability Index; SD, standard deviation; SF-36, Short Form-36 health survey; SF-36 MCS, Short Form-36 health survey – Mental Component Summary; SF-36 PCS, Short Form-36 health survey – Physical Component Summary.

### Discriminant validity

Results of the discriminant analysis are summarized in Table 3 (paid work) and Table 4 (productivity within the home). Employed subjects with lower physical functioning at baseline, as assessed by the HAQ-DI, had a significantly higher burden of disease in their productivity in the workplace compared with subjects with higher physical functioning. Subjects in the 'worst' health group reported increased absenteeism (3.0 versus 1.1 mean days missed; *P *≤ 0.001) and presenteeism (6.8 versus 3.4 mean days with reduced productivity at work; *P *≤ 0.001) compared with patients in the 'best' health group.

**Table 3:** WPS-RA baseline responses by HAQ-DI and SF-36: work productivity of employed subjects in the modified intent-to-treat population

Instrument^a^	Number of days of work missed over the previous month, mean (SD)		Number of days with productivity ≤ 50% at work over the previous month, mean (SD)		Rate of arthritis interference with WP^b ^over the previous month, mean (SD)	
			
	Worst	Best	Worst	Best	Worst	Best
HAQ-DI (cutoff 0.5 and 1.5)	3.0 (7.19)	1.1^c ^(3.45)	6.8 (8.96)	3.4^c ^(6.88)	4.8 (2.77)	4.4^d ^(2.67)
	n = 46	n = 35	n = 46	n = 35	n = 46	n = 35
						
SF-36 PCS (cutoff 21.76 and 35.26)	2.9 (6.96)	1.3^e ^(4.50)	8.7 (10.02)	2.2^c ^(4.60)	6.0 (2.13)	3.5^c ^(2.16)
	n = 21	n = 20	n = 21	n = 20	n = 21	n = 20
						
SF-36 MCS (cutoff 38.36 and 54.67)	4.1 (8.07)	0.7^c ^(1.59)	10.6 (11.07)	4.0^c ^(7.83)	5.2 (2.89)	4.1^c ^(2.47)
	n = 20	n = 21	n = 20	n = 21	n = 20	n = 21

^a^Cutoff points represent first and third quartiles of baseline scores; 'worst' group (HAQ-DI score ≥ third quartile; SF-36 score ≤ first quartile) and 'best' group (HAQ-DI score ≤ first quartile, SF-36 ≥ third quartile). ^b^Score on a scale of 0 to 10 points (0 = no interference and 10 = complete interference). WPS-RA recall period is 1 month. ^c^*P *value ≤ 0.001, ^d^*P *value < 0.01, ^e^*P *value ≤ 0.05 best versus worst; *P *values were obtained using the non-parametric bootstrap-t method. HAQ-DI, Health Assessment Questionnaire – Disability Index; SD, standard deviation; SF-36, Short Form-36 health survey; SF-36 MCS, Short Form-36 health survey – Mental Component Summary; SF-36 PCS, Short Form-36 health survey – Physical Component Summary; WP, work productivity; WPS-RA, Work Productivity Survey – Rheumatoid Arthritis.

The WPS-RA was able to discriminate between patients with lower versus higher physical or mental HRQoL, as assessed by the SF-36 PCS and MCS scores. Employed subjects with lower PCS scores missed significantly more days of paid work (2.9 versus 1.3 mean days; *P *≤ 0.05) and had more days with reduced productivity while at work (8.7 versus 2.2 mean days; *P *≤ 0.001) compared with subjects with higher PCS scores. The reported disease interference in terms of physical HRQoL with work productivity was significantly higher in subjects in the 'worst' health group compared with those in the 'best' health group (6.0 versus 3.5 mean rate on a scale of 0 to 10; *P *≤ 0.001). The findings were similar when evaluating MCS scores (Table 3).

A similar pattern was seen when examining the differences in responses to the WPS-RA home productivity-related questions for all subjects. Household activity and social activity limitations were significantly higher in subjects in the 'worst' compared with the 'best' health groups for HAQ-DI, PCS, and MCS (Table 4). Subjects with higher physical functioning or PCS or MCS missed fewer days of household activities and leisure activities and had fewer days with reduced productivity in their home activities compared with those with lower physical functioning or HRQoL. Consistent with the quantitative results, those with higher scores in physical functioning or HRQoL also reported significantly lower interference of RA with their home productivity. The interference scores for household work tended to be higher than the interference scores for paid work for the 'worst' groups. The household scores were typically at least 7.0 on a scale of 0 to 10 (where 10 is complete interference), whereas the workplace rates ranged from 4.8 to 6. The 'best' groups for both household and paid work tended to report scores next to or below the average rate of interference (5 on a scale of 0 to 10, where 0 is no interference).

**Table 4:** WPS-RA baseline responses by HAQ-DI and SF-36: home productivity and daily activities of all randomly assigned subjects in the modified intent-to-treat population

Instrument^a^	Number of days of household work missed over the previous month, mean (SD)		Number of days with household productivity ≤ 50% over the previous month, mean (SD)		Number of days of missed family, social, or leisure activities over the previous month, mean (SD)		Number of days with outside help over the previous month, mean (SD)		Rate of arthritis interference with household WP^b ^over the previous month, mean (SD)	
					
	Worst	Best	Worst	Best	Worst	Best	Worst	Best	Worst	Best
HAQ-DI (cutoff 0.75 and 1.75)	12.5 (10.79)	6.4^c ^(8.01)	14.0 (10.35)	9.4^c ^(9.53)	5.1 (7.80)	3.6^c ^(6.56)	1.5 (4.90)	1.1 (4.57)	7.0 (2.42)	5.1^c ^(2.82)
	n = 116	n = 96	n = 115	n = 96	n = 116	n = 96	n = 114	n = 95	n = 113	n = 95
										
SF-36 PCS (cutoff 21.98 and 33.0)	13.7 (10.91)	3.4^c ^(5.13)	14.7 (11.21)	6.2^c ^(7.06)	5.7 (7.94)	1.7^c ^(3.26)	1.4 (4.72)	0.5^c ^(1.24)	7.0 (2.77)	4.0^c^ (2.52)
	n = 55	n = 52	n = 54	n = 52	n = 55	n = 52	n = 55	n = 51	n = 55	n = 51
										
SF-36 MCS (cutoff 35.31 and 54.07)	14.0 (11.05)	4.7^c ^(6.16)	15.9 (10.13)	5.8^c ^(7.69)	6.9 (8.24)	0.6^c ^(1.19)	1.5 (4.74)	0.8 (4.20)	7.1 (2.51)	4.1^c ^(2.73)
	n = 53	n = 53	n = 53	n = 53	n = 53	n = 53	n = 52	n = 52	n = 52	n = 52

^a^Cutoff points represent first and third quartiles of baseline scores; 'worst' group (HAQ-DI score ≥ third quartile; SF-36 score ≤ first quartile) and 'best' group (HAQ-DI score ≤ first quartile, SF-36 ≥ third quartile). ^b^Score on a scale of 0 to 10 points (0 = no interference and 10 = complete interference). WPS-RA recall period is 1 month; ^c^*P *value ≤ 0.001 best versus worst; *P *values were obtained using the non-parametric bootstrap-t method. HAQ-DI, Health Assessment Questionnaire – Disability Index; SD, standard deviation; SF-36, Short Form-36 health survey; SF-36 MCS, Short Form-36 health survey – Mental Component Summary; SF-36 PCS, Short Form-36 health survey – Physical Component Summary; WP, work productivity; WPS-RA, Work Productivity Survey – Rheumatoid Arthritis.

The WPS-RA was able to discriminate the 'worst' and 'best' health groups in all home-related questions, with the exception of 'number of days with outside help'. Differences between the two groups were less than 1 day, on average. It should be noted that, at baseline, 'days with outside help' were reported by only 18% of the subjects.

Consistent results were obtained for the eight SF-36 domains (data not shown), showing that the WPS-RA was able to discriminate patients with lower and higher HRQoL. All group differences were statistically significant, except in the 'number of missed days of work' (for the physical functioning domain) and in the 'number of days with outside help' for the bodily pain and mental health domains.

### Responsiveness and reliability

#### WPS-RA changes from baseline by ACR20 response at week 24

**Figure 1:**
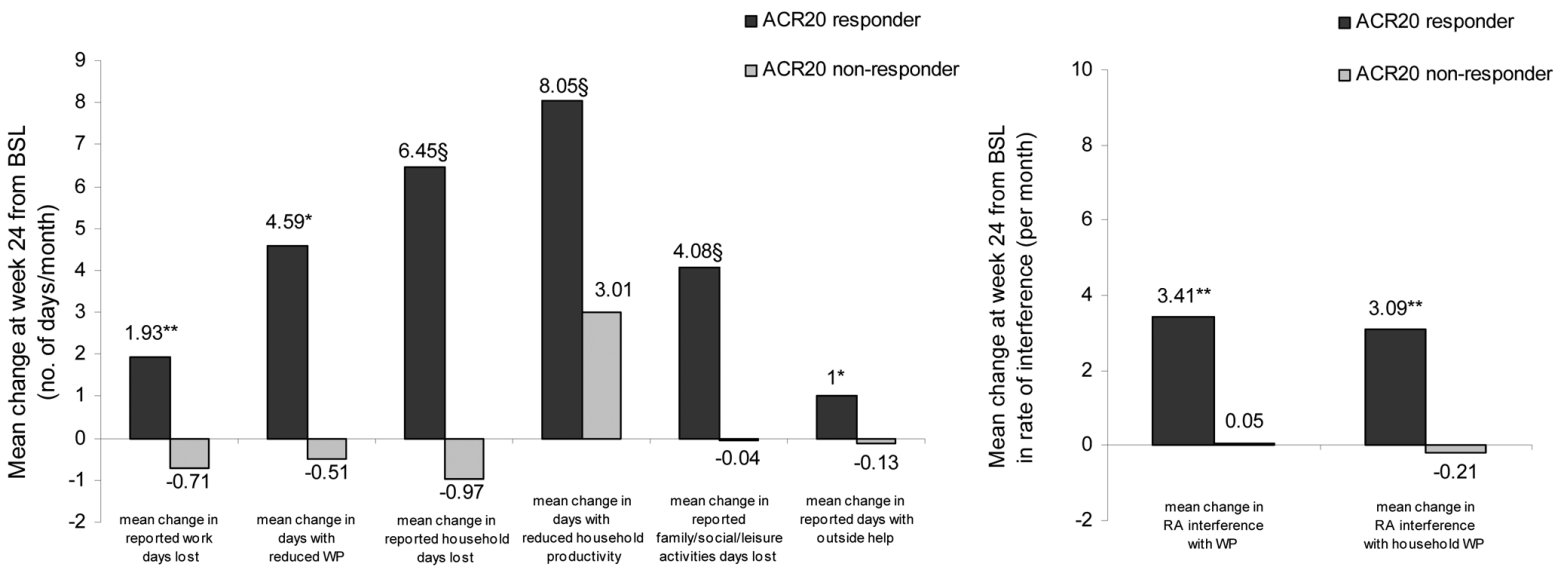
Change from baseline in Work Productivity Survey – Rheumatoid Arthritis (WPS-RA) by American College of Rheumatology 20% improvement criteria (ACR20) clinical response at week 24. ^§^*P *≤ 0.001, **P < 0.01, *P ≤ 0.05 responders versus non-responders; *P *values were obtained using the non-parametric bootstrap-t method. Rate of interference is a score on a scale of 0 to 10 points (0 = no interference and 10 = complete interference). WPS-RA recall period is 1 month. BSL, baseline; RA, rheumatoid arthritis; WP, work productivity.

The improvements in productivity within and outside the home were significantly higher in patients who achieved an ACR20 response at week 24 compared with those who did not (regardless of treatment assignment) (Figure 1). On average, employed ACR20 responders reported higher reductions in absenteeism (1.93 days per month) and larger decreases in days with reduced productivity at work (4.59 days per month) compared with non-responders who reported increases (that is, worsening) in both absenteeism and presenteeism. Even further reductions in lost productivity at home and in participation in daily activities were reported by ACR20 responders. ACR20 responders reported significantly fewer days lost in terms of household work (7.4 fewer days lost per month) and leisure activities (4.1 fewer days) compared with non-responders.

**Figure 2:**
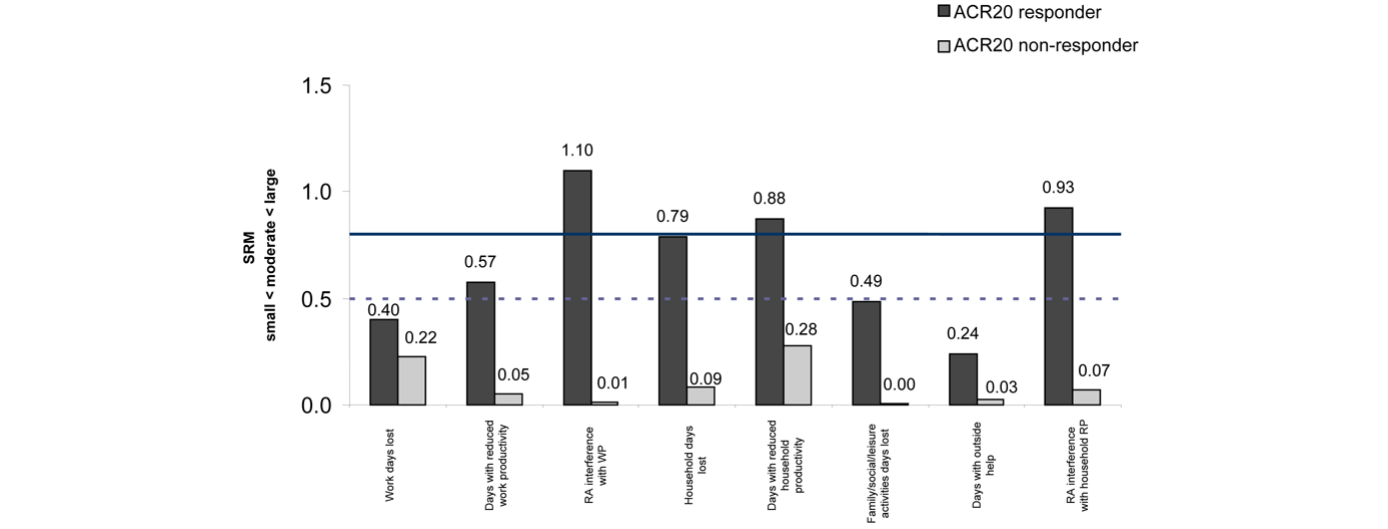
Standardized response mean (SRM) of changes from baseline in Work Productivity Survey – Rheumatoid Arthritis by American College of Rheumatology 20% improvement criteria (ACR20) clinical response at week 24. SRM is small below the dashed line (0.5), moderate between the two lines, and large above the solid line (0.8).

When the variability of measurement was taken into account, the mean changes in productivity within and outside the home in the ACR20 non-responder group were small (SRM < 0.3) (Figure 2). In comparison, ACR20 responders experienced moderate and large mean changes in productivity relative to their standard deviations, with the exceptions of absenteeism and days with hired outside help, where the effect of change was small (SRM = 0.4 and 0.24, respectively).

#### WPS-RA changes from baseline by HAQ-DI response at week 24

**Figure 3:**
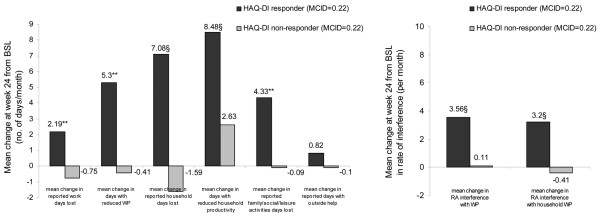
Change from baseline in Work Productivity Survey – Rheumatoid Arthritis (WPS-RA) by Health Assessment Questionnaire – Disability Index (HAQ-DI) response at week 24. ^§^*P *≤ 0.001, **P < 0.01 responders versus non-responders; *P *values were obtained using the non-parametric bootstrap-t method. Rate of interference is a score on a scale of 0 to 10 points (0 = no interference and 10 = complete interference). WPS-RA recall period is 1 month. Response is defined as a decrease from weeks 0 to 24 in the HAQ-DI score of greater than or equal to the minimum clinically important difference (MCID) in absolute value. BSL, baseline; RA, rheumatoid arthritis; WP, work productivity.

**Figure 4:**
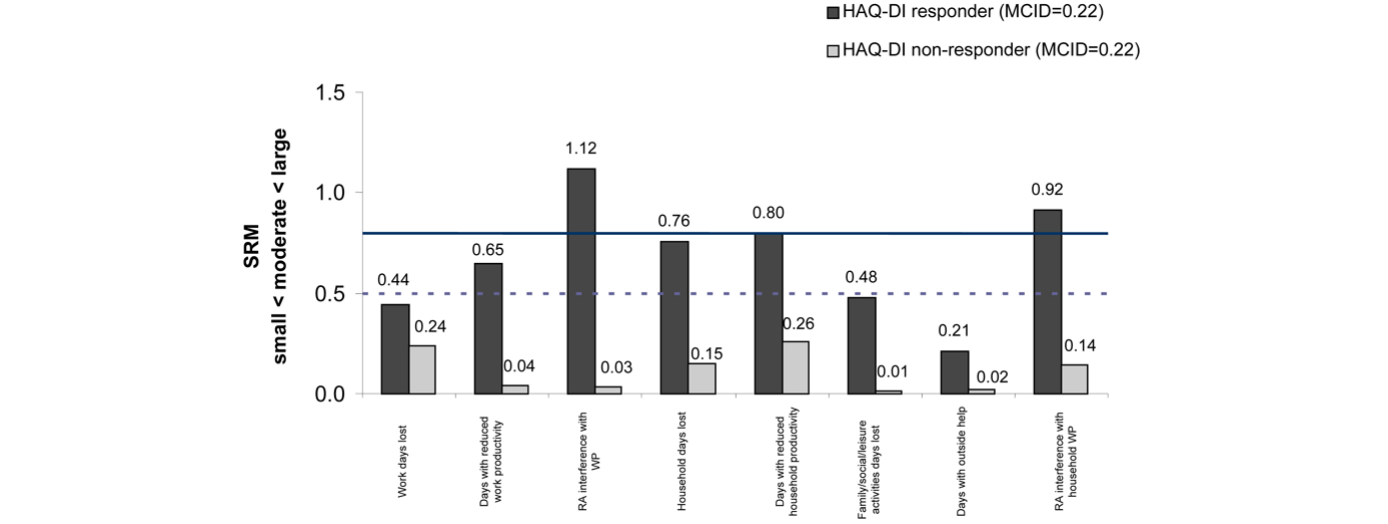
Standardized response mean (SRM) of changes from baseline in Work Productivity Survey – Rheumatoid Arthritis by Health Assessment Questionnaire – Disability Index (HAQ-DI) response at week 24. SRM is small below the dashed line (0.5), moderate between the two lines, and large above the solid line (0.8). Response is defined as a decrease from weeks 0 to 24 in the HAQ-DI score of greater than or equal to the minimum clinically important difference (MCID) in absolute value.

The WPS-RA demonstrated responsiveness to clinically meaningful changes in HAQ-DI, as defined by an MCID of 0.22 (Figures 3 and 4). It also showed reliability, as similar findings were achieved with the ACR20 clinical change.

Sensitivity analysis was conducted to test the responsiveness of the WPS-RA in patients completing the study (data not shown). Of the 220 patients randomly assigned, 104 were still present in the study at week 24. Responders completing the study reported higher improvements compared to non-responders, showing similar trends to the ones presented in the Result section with higher improvements in responders completing the study compared with non-responders. However, due to small sample sizes, statistically significant differences were not attained in all WPS-RA questions.

## Discussion

The objective of this paper was to evaluate the initial psychometric properties of the WPS-RA as a tool to estimate productivity limitations due to RA in the workplace and in household activities. In so doing, we sought to demonstrate that the WPS-RA could efficiently evaluate both the impact of the disease and clinical interventions on work outcomes in patients with RA. To this end, the discriminant validity, the responsiveness to clinical changes, and the reliability of the survey were evaluated in subjects enrolled in a clinical trial for the treatment of active RA.

OMERACT (Outcome Measures in Rheumatology) is an international, informal network of clinicians and scientists interested in outcome measurement across the spectrum of rheumatology intervention studies. OMERACT meetings 6 and 7 have highlighted the importance to patients of consideration of the impact of RA on paid and unpaid work outcomes as they represent an important component of the health and well-being of RA patients [15,29,30]. Patient-reported outcomes (PROs) in RA have long been included in RA trials as they capture the patient's perspective of the disease process and the impact of treatments on the disease. Well-accepted PRO measures used in RA clinical trials include the HAQ-DI (which measures functional disability), the SF-36 (a generic HRQoL measure), and various pain assessments. The impact of RA on work outcomes is not currently a core component of RA clinical trials. We have thus taken initial steps to create an assessment for use in clinical trials, designed to efficiently capture the impact of RA and its treatment on work outcomes, broadly defined to include both paid and unpaid work. During the recent OMERACT 9 meeting, based on the available filter evidence (truth, discrimination, and feasibility) [31], the WPS-RA was one of six instruments identified by the OMERACT Worker Productivity group as a possible candidate for assessing productivity changes in RA. OMERACT 9 proceedings are expected to be published this year and will fully describe the findings from the latest meeting.

In capturing work absences due to arthritis, we considered both full and partial days (that is, days of work missed and days with productivity reduced by at least half). Kessler and colleagues [32] have used the term 'work cut back and work loss days', whereas others have used the National Health Interview Survey (NHIS) approach of disability days and partial days in bed [33]. Still others have used work loss days and days worked but with productivity reduced by half or more [34]. Similar subjective assessments of perceived effectiveness (or lack thereof) in performing work activities have been taken in other chronic disease states such as migraine headache and depression [35-37]. Responses tend to be based on the patient's estimate of completely missed work days and of days that they worked but their productivity was reduced. Previous assessments have asked patients to estimate their productivity at work when working with symptoms and asked the patients to estimate their productivity on a scale of 0 to 100. However, it was felt that asking respondents to estimate the days in which they were less than 50% productive allowed for easier responses that were as meaningful. Lerner and Lee [38] have noted that respondents generally underestimate time lost, so this would be a more conservative estimate of work productivity.

The discriminant validity of the WPS-RA was evaluated relative to a standard measure of physical functioning (HAQ-DI) and a validated generic HRQoL measure (SF-36). Subjects with lower physical functioning or HRQoL scores tended to have statistically greater productivity losses due to RA within and outside the home compared with subjects with higher scores; 83 of the 88 validation evaluations of the WPS-RA were statistically significant, showing that the survey has properties supportive of discriminant validity.

The known groups used to assess discriminant validity were constructed using the first and third quartiles of the instrument scores at baseline. If clinically meaningful thresholds instead of the first and third quartiles were used for physical disability or HRQoL, this would have led to a comparison of unbalanced groups for the validity analysis. However, recognized clinical thresholds were considered to assess the responsiveness of the WPS-RA, in support of the discriminant validity.

The responsiveness of the WPS-RA was tested against two meaningful clinical changes: the ACR20 and the HAQ-DI responses. At week 24, both ACR20 and HAQ-DI responders reported significant reductions in lost productivity within and outside the home, whereas non-responders reported mainly a worsening in their productivity. The effect size for productivity changes in ACR20 or HAQ-DI responders was moderate to large for six of eight WPS-RA questions (SRM = 0.48 to 1.12). In non-responders, the magnitude of change was negligible (SRM < 0.1) or small (SRM < 0.3). These results demonstrate the responsiveness of the survey, given the differences in effect size seen for responders and non-responders and the similarities in responsiveness for both criteria (ACR and HAQ).

The WPS-RA is interviewer-administered, is based on patient self-report, and has a 1-month recall period. The limitations of self-report data have been acknowledged, but previous work comparing self-report data to 'objective' data from work records and diaries supports the value of self-report data as being efficient, reasonably accurate, and often the only means by which such information can be collected [38-41]. Health-related work productivity questionnaires vary in the length of recall time, and there is no consensus regarding the ideal reporting period [38]. A 1-month recall is considered sufficient to be likely to capture events and does not overly burden respondents with too great a frequency of question-asking (as a daily diary might).

We will be undertaking future work to develop a self-administered version of the WPS-RA and to assess the utility of the instrument outside clinical trials and consequently assess criterion validity. Given the nature of the questions and the relatively good completion rates in the trial, we would expect no major differences between the self-administered and interviewer-administered versions. Criterion validity was not assessed at this point since such an assessment explores the relationship between self-report and objective productivity measures and thus determines whether responses are related substantially to actual output. This type of assessment would be more appropriate within specific workplace studies. The clinical relevance and generalizability of WPS-RA results outside of clinical trials will be assessed by defining norms for the MCID of each of the questions of the instrument.

## Conclusions

The WPS-RA survey was found to be a valid instrument, able to discriminate between patients with different RA symptom severity, and responsive to recognized clinical changes. The survey can capture the impact of active RA and its treatment on important aspects of patients' work, both within and outside the home, and it can be used in clinical trials for the treatment of RA.

## Abbreviations

ACR: American College of Rheumatology; ACR20: American College of Rheumatology 20% improvement criteria; FAST 4WARD: efficacy and safety of certolizumab pegol monotherapy every 4 weeks dosage in rheumatoid arthritis; HAQ-DI: Health Assessment Questionnaire – Disability Index; HRQoL: health-related quality of life; MCID: minimum clinically important difference; MCS: Mental Component Summary (of the Short Form-36 health survey); mITT: modified intent-to-treat; NHIS: National Health Interview Survey; OMERACT: Outcome Measures in Rheumatology; PCS: Physical Component Summary (of the Short Form-36 health survey); PRO: patient-reported outcome; q4w: every 4 weeks; RA: rheumatoid arthritis; SF-36: Short Form-36 health survey; SRM: standardized response mean; WPS-RA: Work Productivity Survey – Rheumatoid Arthritis.

## Competing interests

This paper was funded by UCB Pharma, which sponsored the clinical trial in which the data were collected. JTO is a paid consultant of UCB SA. OP and LR are both employed full-time by Global Health Outcomes Research, UCB Pharma. The three people listed in the Acknowledgments also work full-time for UCB Pharma.

## Authors' contributions

JTO helped create the WPS-RA and wrote the Introduction and helped with the Discussion section of the manuscript. OP participated in the conceptualization and statistical analysis and in the writing and review of the manuscript. LR wrote sections of the manuscript and reviewed it. All authors read and approved the final manuscript. The authors acknowledge Yves Brabant, who provided statistical programming support, and Martin Brown and Lucian Ionescu, who both reviewed the manuscript and provided constructive comments to improve it.

## Supplementary Material

Additional file 1 A copy of the WPS-RA questionnaire. Click here for file
