# Supplementary material for: Discriminant validity, responsiveness and reliability of the rheumatoid arthritis-specific Work Productivity Survey (WPS-RA)
Source: Arthritis Res Ther. 2009 May 20;11(3):R73. doi: 10.1186/ar2702 (PMC2714119; doi:10.1186/ar2702)
Supplement: Additional file 1 — A copy of the WPS-RA questionnaire. [file ar2702-S1.doc]

# RA specific Work Productivity Survey (WPS-RA)

Site staff should obtain the following information from the patient.

1. Is patient currently employed outside of home?

Yes - continue

No - go to question 1b.

a**. If yes**,

(1) Indicate their occupation: _______________

(2) Check the box that best describes their job function from the list below:

Non-manual (administrative, managerial, supervisory, office and other professional, such as teacher)

Mixed, non-manual and manual (sales and service occupations such as waitress, personal care attendant, patient care nurse, nurse’s aide, driver)

Manual with no supervisory duties (carpenter, roofer, loader)

GO TO QUESTION 2

b**. If no**, (patient is not currently employed outside of home) please check the box that best describes their status:

Homemaker

Retired

Student

Unable to work due to arthritis

Unable to work due to non-arthritis health problems

Other (i.e. perform volunteer work)

GO TO QUESTION 5

2. How many days in the last month did the patient miss work because of arthritis? (If none, please write 0) ___ days*

3. How many days in the last month was the patient’s productivity at work reduced by half or more because of arthritis? (Do not include days counted in question 2) (If none, please write 0) ___ days*

4. In the last month, how much has arthritis interfered with the patient’s work productivity (work outside of home) on a scale of 0-10, where 0 = "no interference" and 10 = "complete interference"? _____

ASK ALL PATIENTS

5. How many days in the last month did the patient not do household work because of arthritis (If none, please write in 0)? ___ days*

6. How many days in the last month was productivity in household work reduced by half or more because of arthritis? (Do not include days counted in question 5) (If none, please write 0) ___ days*

7. How many days in the last month did the patient miss family, social or leisure activities because of arthritis (If none, please write in 0)? ___ days*

8. How many days in the last month did the patient have to hire outside help (i.e. housekeeper) because of arthritis (If none, please write in 0)? ___ days*

9. In the last month, how much has arthritis interfered with the patient’s household work productivity on a scale of 0-10, where 0 = "no interference" and 10 = "complete interference"? _____

*Do not count days missed due to scheduled per protocol study visits.

**This questionnaire is used by UCB under a copyright license from Pharmacia/Pfizer.  It is protected by copyright and it may not be further copied by any means without permission, save as may be permitted by law.**
